# *Me’akai* in Tonga: Exploring the Nature and Context of the Food Tongan Children Eat in Ha’apai Using Wearable Cameras

**DOI:** 10.3390/ijerph16101681

**Published:** 2019-05-14

**Authors:** Loma Veatupu, Viliami Puloka, Moira Smith, Christina McKerchar, Louise Signal

**Affiliations:** Health Promotion & Policy Research Unit, University of Otago, Wellington 6242, New Zealand; 02llveatupu@gmail.com (L.V.); viliami@hauora.co.nz (V.P.); moira.smith@otago.ac.nz (M.S.); christina.mckerchar@otago.ac.nz (C.M.)

**Keywords:** Tonga, food, diet, children

## Abstract

Unhealthy food consumption is a key driver of the global pandemic in non-communicable diseases (NCDs). The Government of Tonga has prioritised NCD prevention due to the very high rates of NCDs in the Kingdom. This research examines the nature and context of the *me’akai* (food) consumed by Tongan children in Ha’apai using wearable cameras. Thirty-six randomly selected 11-year-old children used wearable cameras to record their lives for three days, as part of the wider Kids’Cam Tonga project. Images were analysed to assess the participants’ food consumption according to a new data analysis protocol for Tonga. Core foods were defined as including breads and cereals, fresh fruit, vegetables, meat and alternatives, and staple vegetables. Non-core food types included confectionery, unhealthy snack foods, edible ices, and processed meat. Tongan researchers led the research in partnership with the Government of Tonga. Overall, children were observed to have consumed a mean of 4.5 (95% CI 3.3, 6.7) non-core and 2.3 (95% CI 1.8, 2.9) core foods per 10 h day, excluding mixed meals. Unhealthy snack foods, confectionary, and cookies, cakes, and desserts were the most commonly consumed non-core foods, and fresh fruit was the most frequently consumed core food. Snacking was the most frequent eating episode observed, with children snacking on non-core foods four times a day (95% confidence interval (CI) 2.5 to 6.2) compared to 1.8 (95% CI 1.3 to 2.6) core food snacks per day. Most commonly, children were observed eating at home, at school, and on the road while out walking. The most common sources of food were the home, other children, and the supermarket. On average, children consumed one purchased product per day, almost all (90%) of which were non-core. Children were also observed eating an average of just less than one mixed meal per day. Less than half (45.2%) of all mixed meals observed were traditional foods. This research illustrates the presence, and likely dominance, of energy-dense nutrient-poor (EDNP) foods in the diet of these Tongan children. It highlights a transition from a traditional diet and suggests that these children live in an obesogenic environment, one that promotes obesity as a normal response to an abnormal environment. The findings support efforts by the Government of Tonga for the implementation of a healthy School Food Policy, junk food taxes, and initiatives to ban the importation of EDNP foods. This study has relevance for other Pacific Island nations and all nations concerned with addressing obesity and other diet-related NCDs.

## 1. Introduction

Over recent years there has been a substantial rise in non-communicable diseases (NCDs) worldwide [1]. The Kingdom of Tonga is among the countries fighting the NCD pandemic, with 81% of all deaths attributable to NCDs compared to the global average of 70% [1]. The Government of Tonga has recognised the negative health effects and financial burden of NCDs, and therefore has prioritised NCD prevention and control [2].

Obesity is a leading cause of preventable NCD morbidity and mortality [3]. Childhood obesity is very widespread, a leading precursor of adult obesity and associated with the early onset of various NCDs. Evidence shows that 54% and 36% of girls and boys, respectively, in Tonga were either overweight or obese [4]. However, obesity during childhood is preventable and therefore, childhood is a critical period for public health intervention [5].

In Tonga, traditional diets mainly consisted of starchy root produce (e.g., *manioke* (cassava), *ufi* (yam), and *talo* (taro)), fruit (e.g., *niu* (coconut) and *lesi* (pawpaw)), fresh seafood, and green leafy vegetables (e.g., *pele* (spinach) and *lu* (taro leaves)). However, there has been a nutrition transition from traditional diets to a modernised diet of cheap, low quality, highly processed, and high-fat imported foods [6,7]. The consumption of such energy-dense nutrient-poor (EDNP) foods is associated with the development of obesity and diet-related NCDs [6,8].

There appears to be limited literature assessing the diet of Tongan children. What research there is uses self-report measures [9,10], which are prone to limitations such as recall bias and desirability bias [9,11,12]. However, recent studies in the United Kingdom and New Zealand have used wearable cameras to objectively capture the nature and context of food consumption [13,14,15,16,17,18,19], though no studies have explored food consumption in children younger than 13. Wearable cameras provide an effective method to assess food consumption, especially along with 24-h diet recall [14,17]. Cameras also enable an assessment of the environmental and social context that surrounds eating and dietary behaviours [13] including food marketing in children’s environments [16] and opportunities for food and drink purchasing and consumption by teenagers during their journeys between home and school [15].

Kids’Cam is a methodology that uses wearable cameras to assess the world in which children live and their interaction with it [16,18]. The Kids’Cam method has been employed in Tonga [20,21]. This study utilises the Kids’Cam Tonga data to assess the nature and context of the *me’akai* (food) eaten by Tongan children in the outer island of Ha’apai.

## 2. Materials and Methods

### 2.1. Study Design

Kids’Cam Tonga is an observational cross-sectional study. Participants were 108 randomly selected children aged 10–12 years from 16 randomly selected schools: 72 participants from 12 schools on Tongatapu (the main island) in 2016, and 36 from four schools on Ha’apai in 2017. In 2016 the population of Ha`apai numbered 6125 [4].

Participating children wore a wearable camera (Autographer) on lanyards around their neck for three consecutive days (Friday to Sunday). The cameras automatically took wide-angled, 136° photos of the participant’s environment approximately every seven seconds. The Kids’Cam Tonga research team worked collaboratively with the Ministry of Health (MoH), Ministry of Education and Training (MoET) and the Tonga Health Promotion Foundation (TongaHealth) to undertake the research. Full details of the original Kids’Cam study are published elsewhere [16,18].

### 2.2. Ethical Approval

The University of Otago Human Ethics Committee (Health) (13/220) and the Tonga National Health Ethics and Research Committee (#290116) provided ethical approval to study any aspect of the world children live in and their interaction with it. All precautions were taken to ensure that participants were protected from any potential harm. The anonymity of participating children and third parties is protected by obscuring identifying features and faces in any published images.

### 2.3. Sampling and Recruitment

The Tongan MoET provided a list of all schools in Tongatapu and Ha’apai that included a Class 6 roll. Sampling was conducted in two stages: (1) schools were randomly selected by their location based on probability-proportional-to-size sampling methods; and (2) children were randomly selected from each of these schools. Prior to the study, the sample size was determined based on the total number of schools and children that could be covered by the project budget. In Ha’apai, six children were selected from each of three schools and 18 participants from the largest school.

Officials from the Tongan MoET facilitated school recruitment. School principals and/or senior teachers were contacted to explain the study, its purpose, and the data collection procedure. The whole school was informed about the project, as permission was required from the school community to allow the study to take place in the school. Written consent was gained from the principal of each school. Consenting principals invited the selected children and their families to participate and provided them with information sheets and consent forms for both children and their parents to fill out. There was a briefing session at each school a day prior to the start of data collection to explain the project to those children and their parents or guardians who provided written consent.

### 2.4. Data Collection and Management

The children were instructed to wear the camera all day for three consecutive days, from getting up until going to bed, except in situations where privacy is expected (e.g., bathrooms), when undertaking vigorous sport or when asked to remove it. Following data collection, cameras were collected and images downloaded, with children given the opportunity to review and delete any photos they did not want included in the study before the researchers viewed them. To determine body mass index (BMI), each child’s height (m) and weight (kg) were measured using a stadiometer and scales (both calibrated), respectively.

Basic demographic information was also collected, but 24-h food recall was not included due to budgetary constraints. Approved images were downloaded to a password-protected server, saved in secure cloud storage, and backed up to a password-protected external hard drive. There were 300,000 images captured in Ha’apai, out of 1 million images in the whole Kids’Cam Tonga project. Data access is strictly restricted to members of the research team who had signed and agreed to adhere to strict protocols regarding the release and use of data.

### 2.5. Image Content Analysis for the Me’akai Study

Using content analysis, all images from the 36 Ha’apai participants were analysed to assess the participants’ food consumption according to a new data coding protocol for Tonga, Kids’Cam Tonga FOODS [22]. Ha’apai was chosen for analysis as it is an outer island that is less impacted by westernization. The results of this research shed light on the nature and context of food eaten in one of the most traditional areas of Tonga. The coding protocol for core and non-core foods was developed based on proposed Tongan school food policy guidelines [21] and Pacific dietary guidelines [23], consistent with international advice [5], and previous Kids’ Cam coding [16,21]. The foods were coded as either core or non-core, or traditional or non-traditional depending on the nature of the foods being consumed. Core food types included breads and cereals, fresh fruit, vegetables, meat and alternatives, and staple vegetables. Non-core food types included confectionery, unhealthy snack foods (e.g., raw noodles and packet chips), edible ices, processed meat, fruit canned in syrup and sugar cane, fast foods, and cookies, cakes, puddings/desserts and pastries (deep fried and baked). Mixed meals (i.e., mixed ingredients (e.g., pizza, *supo* (soup), chop suey) and/or multiple items on one plate) were coded separately as “non-traditional mixed meal” or “traditional mixed meal” as it was often not possible to determine the exact nutritional profile of the meal from the images. However, it was possible to reliably distinguish traditional from other mixed meals because of the distinct nature of traditional food and the knowledge of the Tongan researchers. The codes are presented in Table 1 and include the food type, eating episode, the setting in which it occurred, food source and the purchaser if purchased. Detailed coding instructions and definitions are included in the protocol [22]. A reliability test was conducted, and coders achieved 90% concurrence with model answers on a test dataset of 100 images before coding commenced.

### 2.6. Statistical Analysis

Statistical analysis was conducted in Stata 14 (StataCorp, College Station, TX, USA). Inferential statistics incorporated elements to consider clustering of children within schools (95% confidence intervals (CI)) using Stata’s svy prefix commands and associated weighting options. Descriptive analysis was used to describe children by gender, age, BMI, and school. Age- and sex-specific BMI cutoffs were used to classify BMI into “underweight” (standardised BMI: <18.5), “healthy” (standardised BMI: 18.5–24.9), “overweight” (standardised BMI: ≥25.0–29.9) and “obese/morbidly obese” (standardized BMI: ≥30) [24]. Rates were estimated for all core and non-core food consumption and by food type, eating episode, setting, source and purchaser using negative binomial regression. The numerator for these rates was the number of times a food item was consumed by each child and the denominator was the total image recording time for each child, with this number subsequently re-scaled as a rate per ten hour day. Mean rates are presented with 95% confidence intervals (95% CI), and as rates per 10 h of recording time (i.e., per day). There were too few observations of traditional and non-traditional mixed meals to conduct an analysis to determine rates by food type, eating episode, setting, source and purchaser. Therefore, for traditional and non-traditional mixed meals, only the proportions of mixed meals that were traditional and non-traditional foods are presented.

## 3. Results

There was a 100% response rate from the schools and participants involved. Ha’apai participants captured approximately 300,000 images. This analysis used data collected from 35 of the 36 participants, as one participant did not collect sufficient data. Table 2 presents the demographic characteristics of the included study participants. The sample consisted of more males (57.1%) than females (42.9%) and had a mean age of 10.7 years (95% CI 10.5, 10.9) (range 10–12.2 years). The mean BMI of the sample was 18.7 kg/m^2^ (95% CI 17.7, 19.7). The majority (79.9%) of the participants were categorised as “healthy” BMI, a minimal percentage (2.9%) were categorised as “underweight”, and the remaining (17.2%) participants were categorised as either “overweight”, “obese”, or “morbidly obese” [24]. Half (51.5%) of the participants attended one primary school and almost equal proportions of the remaining participants went to three other schools (two at 17.1% and 14.3% for the other).

### 3.1. Core versus Non-Core Foods

**Figure 1:**
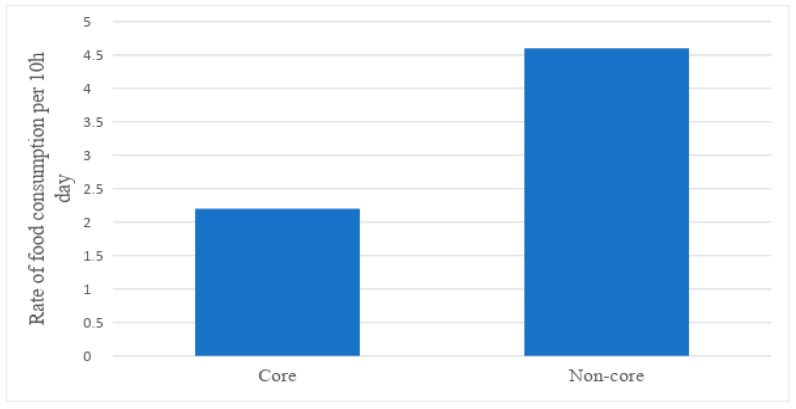
Mean rates of total core and non-core food consumption per 10 h day.

Of all foods that the children were observed consuming over the three days, excluding mixed meals, the majority were non-core foods (67.7%) (Figure 1). Children consumed non-core foods at a mean rate of (4.5 (95% CI 3.3, 6.7)) per day, twice that of core foods (2.3 (95% CI 1.8, 2.9)) (Table 3). Many children were observed sharing their food, particularly when snacking. Thus, they did not always eat the whole food product. However, the images confirm they consumed a portion of it.

#### 3.1.1. Food Type

**Figure 2:**
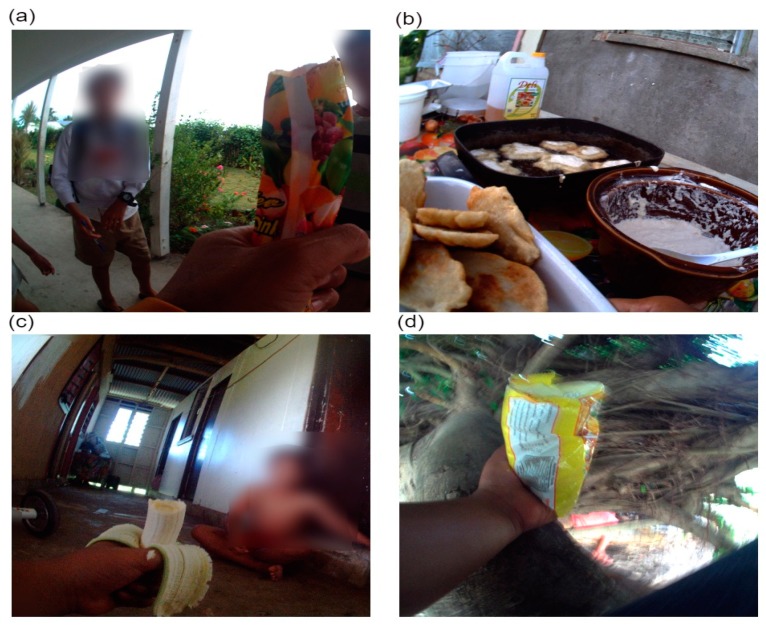
Food types (**a**) non-core edible ice—ice block, (**b**) non-core cookies and cakes—*keke vai* (pancakes), (**c**) core fresh fruit—banana, (**d**) non-core unhealthy snack food—raw noodles.

The greatest proportion of non-core foods was unhealthy snack foods (33.8%), followed by confectionery (25.6%) and cookies, cakes, desserts/puddings and pastries (21.6%) (Table 3 and Figure 2). Children were observed consuming a mean of 1.5 (95% CI 1.1, 2.1) unhealthy snack foods, 1.2 (95% CI 0.8, 1.7) items of confectionery, and 1.0 (95% CI 0.7, 1.4) cookies, cakes, desserts/puddings, and pastries per day. On average, participants consumed an edible ice every second day (0.5 (95% CI 0.1, 1.8)).

Fresh fruit was also a frequently eaten food type per day (1.3 (95% CI 0.6, 2.6)), making up 57.3% of all observed core foods consumed. This was followed by meat and alternatives (0.4 (95% CI 0.1, 1.1)), and staple vegetables (0.3 (95% CI 0.2, 0.7)). However, traditional and non-traditional mixed meals also included vegetables and meat and alternatives. Overall, the most commonly eaten food types were non-core foods.

#### 3.1.2. Eating Episode

Eating episode was defined as “what meal of the day is it? *Kai pongipongi* (breakfast), *Kai ho’atā* (lunch), *Kai efiafi* (dinner) or a snack”. Snacking was the most frequent eating episode (Table 3). On average, children snacked on non-core foods at twice the rate of core food (4.0 (95% CI 2.5, 6.2) and 1.8 (95% CI 1.3, 2.6) per day, respectively). The consumption of both core and non-core foods at breakfast, lunch and dinner, excluding mixed meals, was low. On average, participants consumed substantially less than one core food for each eating episode: breakfast (0.1 (95% CI 0.0, 0.5)), lunch (0.3 (95% CI 0.1, 1.1)), and dinner (0.1 (95% CI 0.0, 0.7)) per day. Further, participants also consumed less than one non-core food on average for each eating episode: breakfast (0.4 (95% CI 0.1, 1.2)), lunch (0.3 (95% CI 0.1, 0.7)), and dinner (0.0 (95% CI 0.0, 0.0)) per day. Virtually all dinner meals were core foods, whereas breakfast and lunch meals, and snacking were predominantly non-core foods.

#### 3.1.3. Setting

**Figure 3:**
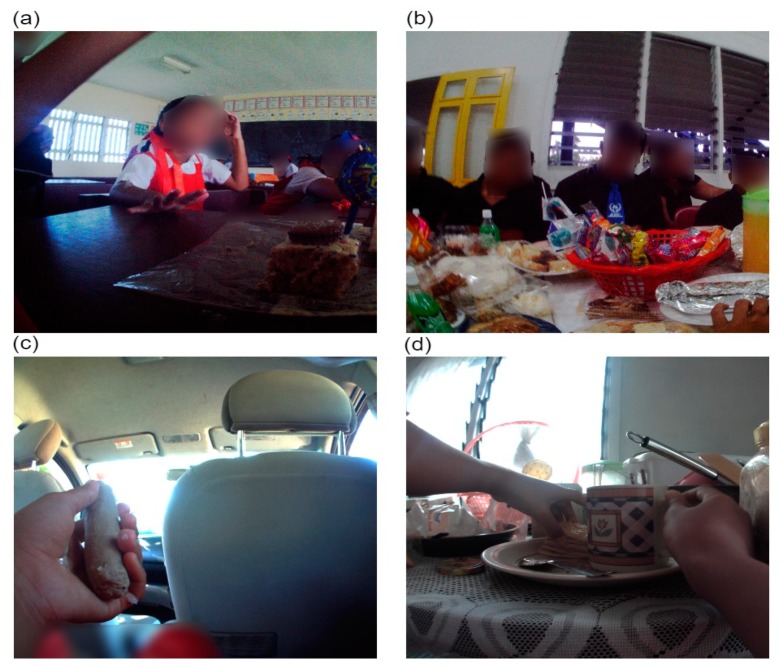
Food consumption by setting (**a**) school, (**b**) church/church hall, (**c**) private transport, and (**d**) home settings.

The consumption of core and non-core foods was most common in the home, at school and road settings (Table 3 and Figure 3). At home, core foods were consumed at a similar mean rate as non-core foods per day (1.3 (95% CI 0.8, 2.1) and 1.6 (95% CI 1.2, 2.0), respectively). At school, children were observed consuming non-core foods at a mean rate 3.5 times that of core foods at school, per day. Furthermore, on the road (most often while out walking) consumption of non-core foods was five times that of core foods.

#### 3.1.4. Source

**Figure 4:**
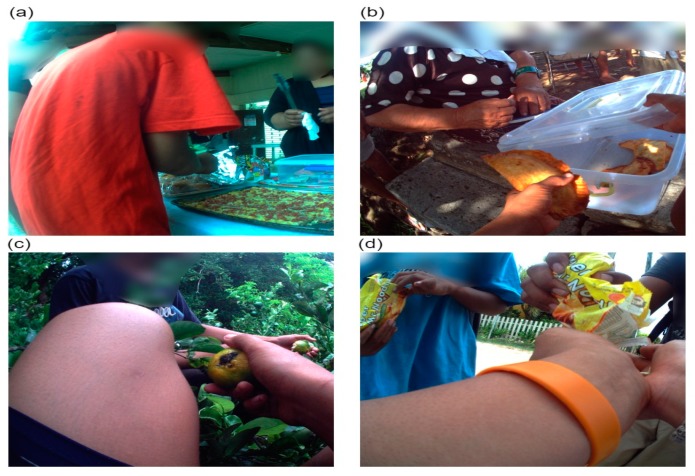
Food source (**a**) market, (**b**) school stall, (**c**) *ngoue’anga* (garden), and (**d**) other child.

On average, the home was the most common source of both core (1.5 (95% CI 1.1, 2.1)) and non-core (1.6 (95% CI 1.2, 2.0)) foods the children were observed consuming per day (Table 3). Other substantial sources of non-core foods were: other children (24.5%), the supermarket (15.3%) and *fale koloa* (10.5%). On average, the participants received and consumed (1.0 (95% CI 0.6, 1.6)) non-core food products from another child every day. Approximately every second day, the children were observed eating a non-core food product sourced from the supermarket (0.6 (95% CI 0.1, 3.2)) and *fale koloa* (0.4 (95% CI 0.0, 4.8)) (Figure 4).

#### 3.1.5. Purchaser

**Figure 5:**
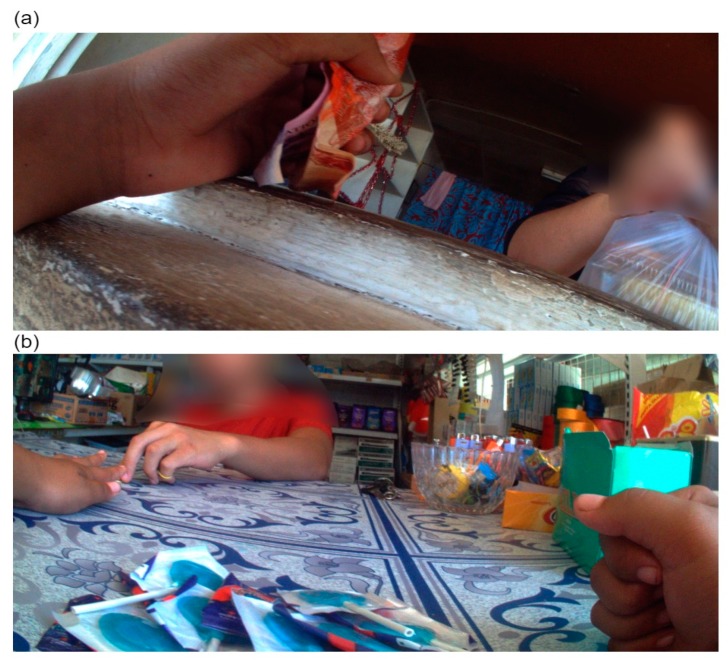
(**a**) and (**b**) are examples of product purchases by the participant.

A product purchase is a food purchased for immediate consumption. On average, children consumed one purchased product per day, nearly all non-core foods (0.9 (95% CI 0.4, 2.2)) (Table 3). Almost all (97.7%) non-core purchases were made by the participants themselves or other children. Very few (2.3%) purchases were made by an adult (Table 3 and Figure 5).

### 3.2. Traditional versus Non-Traditional Mixed Meals

**Figure 6:**
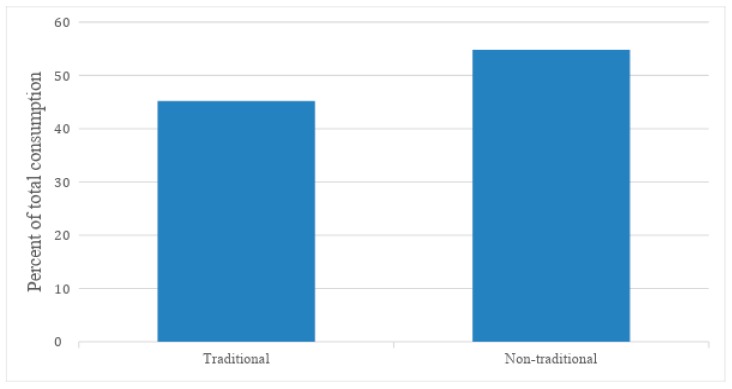
Overall proportion of traditional and non-traditional mixed meal consumption per 10 h day.

**Figure 7:**
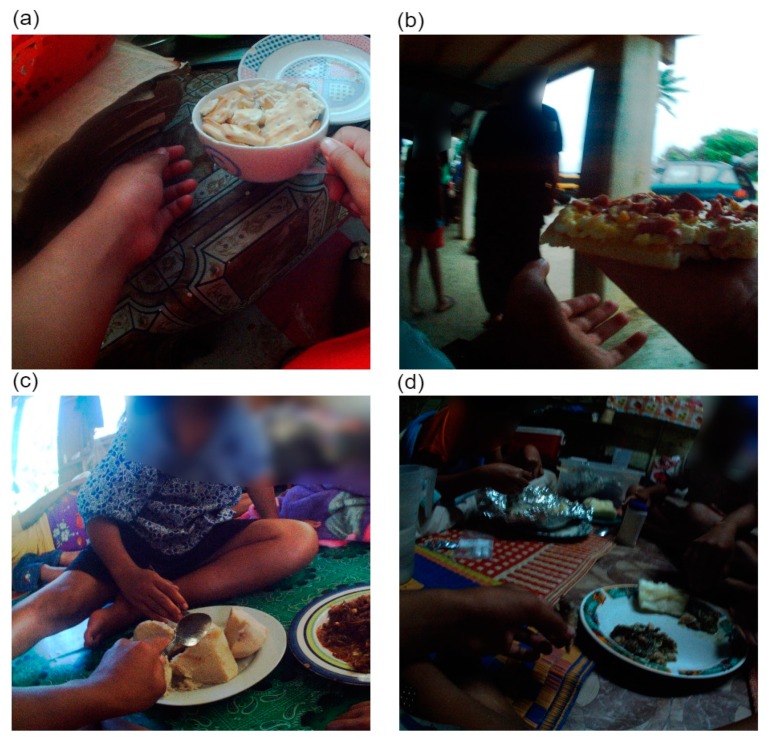
Non-traditional mixed meals (**a**) soaked crackers and (**b**) pizza; and traditional mixed meals (**c**) chop suey and staple vegetables, and (**d**) lu and staple vegetables.

A total of 84 mixed meals, including 17 dinners, were observed being consumed during the three days of data collection. The proportion of mixed meals consumed by the children that were non-traditional was greater than traditional mixed meals (54.8% and 45.2%, respectively) (Figure 6 and Figure 7). Consumption of traditional mixed meals occurred almost always in the home (94.5%), although they were also consumed in the church/church hall (5.5%). Almost three-quarters (71.9%) of all non-traditional mixed meals were eaten in the home, followed by the church/church hall (11.0%) and school (6.5%).

## 4. Discussion

This study reports on the nature and context of the food eaten by Tongan children in the outer island of Ha`apai. It does so by using wearable cameras worn by the child participants. Children were observed consuming non-core foods on average 4.5 times a day, twice that of core foods (2.3), excluding mixed meals. Unhealthy snack foods, confectionary, and cookies, cakes, desserts/puddings and pastries were the most commonly consumed non-core foods, and fresh fruit was the most frequently consumed core food. Snacking was the most frequent eating episode observed, excluding mixed meals, with children snacking on non-core foods four times a day whereas they snacked on core foods nearly twice a day. Most commonly, children were observed eating at home, at school and the road while out walking. At home children ate non-core (1.6 per day) and core (1.3 per day) foods at similar rates. At school they ate non-core foods (1.4 per day) more than three times as often as core foods (0.4 per day). When on the road they ate almost five times as many non-core (0.9 per day) as core foods (0.2 per day). Home was the most common source of children’s food (1.6 non-core and 1.5 core) and the source of the majority of the core food they consumed (83%). The major sources of non-core food were home (40%), other children (25%), the supermarket (15%), and the *fale koloa* (11%). On average, children consumed one purchased product per day, almost all (90%) of which were non-core and which were purchased by the participant or other children (97.7%). Children were also observed eating an average of just less than one mixed meal per day over the three days of data collection, of which nearly all were in the home. Less than half (45.2%) of all mixed meals observed were traditional foods.

To the best of our knowledge, this is the first study internationally to use wearable cameras to document the nature and context of children’s food consumption throughout the day. Previous research on food consumption has been on adults [13], teenagers on the journey to school [15] or children’s drinks consumption [19]. The leadership of Tongan researcher, Viliami Puloka, and the collaboration with the Tongan government ensured the research was conducted in line with the needs and wishes of the Tongan community. As a result, the community embraced the research. There was a 100% response rate from the schools and participants involved and the children captured images of much of their daily lives in the 300,000 images. The images were of a quality that could easily be coded.

However, this methodology does have limitations. While the study shows what the children ate, it has not been possible to reliably record the quantity of the foods eaten. While it was often possible to tell if a child consumed an entire food item, the Tongan children’s habit of sharing food, especially when snacking, made partial consumption of items more difficult to assess. What can be concluded is that the children ate at least a portion of the observed food items. Further, it is likely that some eating episodes have been missed. The recording of only 17 dinners by the 36 children across the three days is inconsistent with the common Tongan practice of eating a family dinner at home, often late every evening. With the children having worn their cameras all day, it is possible that the camera battery needed to be charged by the time they were eating dinner, something the children did overnight. As in this study, Gemming et al. [17] demonstrate the strength of cameras for capturing snacking episodes. However, using cameras in combination with traditional dietary assessment methods, such as 24-h diet recall, provides a stronger evidence base [17]. Had such complementary methods been used in the current study and children enabled to record dinner (with better camera battery life and instructions to keep recording during dinner), it is likely that the quantity of food consumed and any eating episodes missed could have been better assessed. While the study included a random sample of 36 10–12 year-old children from a total population of 6125 people, the study was underpowered. Nevertheless, the findings give considerable insight into the diet of these Tongan children.

There is little literature on the diet of Tongan children, and none that uses wearable cameras. As such, this study makes an important contribution to knowledge about the nature and context of the food that Tongan children eat. There is, however, literature discussing the nutrition transition from a traditional diet to a Western diet dominated by energy-dense nutrient-poor (EDNP) imported foods [6,7]. This study illustrates the presence, and likely dominance, of EDNP foods in the diet of these Tongan children. It highlights a substantial transition from a traditional diet and suggests that, even on the outer island of Ha’apai, Tongan children live in an obesogenic environment, one that promotes obesity as a normal response to an abnormal environment [25]. The research shows the role unhealthy snack foods, confectionary, and cookies, cakes desserts/puddings, and pastries play in snacking in the daily diet of these children, in direct contradiction of nutrition guidance [5,21]. Non-core food is commonly eaten at, or sourced from, home, suggesting that many Tongan families have adopted such food as part of their diet. Children also ate non-core foods at school at more than three times the rate of core foods, despite Tonga having a School Food Policy in place at the time the data was collected [21]. Children commonly eat EDNP food when out walking on the road, possibly in an unsupervised context. Children are purchasing and consuming EDNP food everyday which suggests they have the money to spend, the food is affordable and that it is easily accessed.

Tonga is a Pacific leader in the battle to address NCDs. The Government has had an NCD Prevention Strategy since 2004 [2], they have a multisectoral National NCD Committee, a School Food Policy and hosted the 2016 Pacific NCD Summit that explored how to accelerate progress in addressing the NCD challenge in the Pacific. Key recommendations from the Summit include the need for urgent coordinated action to address time-bound targets, focusing on cost-effective interventions regionally [26]. Tonga is also a global leader in the implementation of sugary drinks and fat and sugary food taxes [27]. This current study provides considerable insight into the challenge faced by Tonga but also offers evidence as to where to intervene.

A key finding of this research is the availability of imported EDNP food in Ha`apai suggesting the need for trade or import restrictions. This may be challenging in the context of global trade and regional trade agreements. However, this study provides evidence of the need for such consideration at regional and international levels so that trade promotes public health and common good [28]. Also, the availability of non-core food in school is an issue. Tonga has struggled to implement the School Food Policy, according to a recent evaluation [21], likely due in part to resource constraints. This current study emphasises the important contribution such a policy could make to the diet of Tongan children if it was well supported. School could be a place for the promotion and provision of healthy food, including healthy traditional, foods. This could occur through requiring only healthy food in schools, not allowing children to leave school at lunchtime and/or provision of food in schools, links to the school curriculum, and utilising school gardens [21]. The affordability of unhealthy food products to children suggests Tonga should increase, and potentially extend, its fat and sugary food taxes, particularly given the evidence of the effectiveness of the Mexican junk food tax [29]. Families could also consider restricting the money children have to spend on such items. Finally, this study reinforces the need for a national conversation about actions to meet the challenge of NCDs, to avoid the consumption of EDNP imported food, and place focus on the traditional diet based on fishing, farming, and gardening.

## 5. Conclusions

Tongan children on Ha’apai eat a diet likely dominated by EDNP food, considerably different from the traditional Tongan diet [6,7]. They appear to live in an obesogenic environment [25] where EDNP *me’akai* is easily accessible and affordable. Urgent action is needed if Tonga is to successfully deliver on its NCD Prevention Strategy [2]. Areas for action suggested by this research include restricting imports of EDNP food, the implementation of the School Food Policy, increased food taxes, and a continuing national conversation about ways to refocus on a healthy diet. It is likely that the findings of this research will provide valuable insights for other Pacific Island nations and all countries struggling with the challenge of the epidemic of NCDs.

## Figures and Tables

**Table 1:** Kids’Cam Tonga FOODS coding schedule.

Food Type	Eating Episode	Setting	Source	Purchaser
**Core**	*Kai pongipongi* (breakfast)	Church/church hall	Adult	Other child
Breads and cereal	*Kai ho’atā* (lunch)	Community venue	Child	Other adult
Fresh fruit	*Kai efiafi* (dinner)	*Fale koloa* (convenience store)	*Fale koloa* (convenience store)	Own purchase
Meat and alternatives	Snacking	Fast food restaurant	Fast food restaurant	Not applicable
Milk products	Unsure	Full service restaurant	Full service restaurant	Unidentified
Staple vegetables				
Vegetables		Home	Home	
		*Ngoue’anga* (garden)	Mobile food vendor	
**Non-core**		Other retail	*Ngoue’anga* (garden)	
Confectionery		Outdoor recreation space	School stall/canteen	
Cookies and cakes		Private transport	Street stall	
Edible ices		Public transport—facility	Supermarket	
Fast foods		Public transport—vehicle	Vending machine	
Fruit canned in syrup, sugar cane		Road	Unknown	
Processed meat		School		
Unhealthy snack foods e.g., packaged chips		Supermarket		
		Uncertain		
**Mixed Meals**				
Traditional mixed meal Non-traditional mixed meal				
**Undetermined**				

**Table 2:** Sociodemographic characteristics of participants in Kids’Cam Tonga FOODS study.

Sociodemographic Variable	Group	*N*	%
Gender	Male	20	57.1
	Female	15	42.9
Age (years) *	10	25	73.5
	11	7	20.6
	12	2	5.9
Body mass index category **	Underweight	1	2.9
	Healthy	28	79.9
	Overweight	5	14.3
	Obese/morbidly obese	1	2.9
School	1	18	51.5
	2	6	17.1
	3	6	17.1
	4	5	14.3

* Information missing for 1 participant; ** categories based on International Obesity Taskforce age- and sex-specific cut-offs (underweight <18.5, healthy 18.5–24.9, overweight 25–29.9, and obese/morbidly obese ≥30) [24].

**Table 3:** Mean rates of core and non-core food consumption per 10 h day, and proportion of total category, by food type, eating episode, setting, source, and purchaser.

Category	Core Foods		Non-Core Foods	
	Rate (95% CI)	% of Category	Rate (95% CI)	% of Category
**Food type**				
Fruits	1.3 (0.6, 2.6)	57.3		
Meat and alternatives	0.4 (0.1, 1.1)	16.5		
Staple vegetables	0.3 (0.2, 0.7)	15.1		
Breads and cereals	0.2 (0.1, 0.3)	7.1		
Vegetables	0.1 (0.0, 0.6)	4.0		
Unhealthy snack foods			1.5 (1.1, 2.1)	33.8
Confectionery			1.2 (0.8, 1.7)	25.6
Cookies and cakes *			1.0 (0.7, 1.4)	21.6
Edibles ices			0.5 (0.1, 1.8)	11.0
Processed meat			0.2 (0.1, 0.5)	4.0
Other fruits			0.2 (0.0, 2.0)	4.0
Fast food			0.0 (0.0, 0.0)	0.0
TOTAL	2.3 (1.8, 2.9)	100.0	4.5 (3.3, 6.7)	100.0
**Eating episode**				
Breakfast	0.1 (0.0, 0.5)	4.5	0.4 (0.1, 1.2)	8.0
Lunch	0.3 (0.1, 1.1)	11.1	0.3 (0.1, 0.7)	5.9
Dinner	0.1 (0.0, 0.7)	4.0	0.0 (0.0, 0.0)	0.0
Snack	1.8 (1.3, 2.6)	80.4	4.0 (2.5, 6.2)	86.1
TOTAL	2.3 (1.8, 2.9)	100.0	4.6 (3.3, 6.7)	100.0
**Setting**				
Home	1.3 (0.8, 2.1)	60.8	1.6 (1.2, 2.0)	34.1
School	0.4 (0.2, 1.1)	18.4	1.4 (0.6, 3.2)	30.2
Road	0.2 (0.0, 1.5)	8.3	0.9 (0.0, 1.9)	19.9
Food retail **	0.0 (0.0, 0.1)	0.9	0.3 (0.1, 0.6)	5.5
Church/church hall	0.1 (0.0, 1.0)	6.5	0.2 (0.0, 0.9)	3.9
Private transport	0.0 (0.0, 0.0) ***	0.0	0.1 (0.0, 0.4)	2.8
Outdoor recreation space	0.0 (0.0, 0.3)	0.9	0.1 (0.0, 0.4)	2.4
Garden	0.1 (0.3, 0.1)	4.2	0.1 (0.0, 2.2)	1.1
TOTAL	2.2 (1.8, 2.9)	100.0	4.6 (3.3, 6.7)	100.0
**Source**				
Home	1.5 (1.1, 2.1)	82.5	1.6 (1.2, 2.0)	39.5
Child	0.2 (0.1, 0.6)	8.7	1.0 (0.6, 1.6)	24.5
Supermarket	0.0 (0.0, 0.0)	0.0	0.6 (0.1, 3.2)	15.3
*Fale koloa*	0.0 (0.0, 0.7)	1.1	0.4 (0.0, 4.8)	10.5
School stall	0.0 (0.0, 0.0)	0.0	0.2 (0.0, 1.3)	4.6
Adult	0.0 (0.0, 0.0)	0.0	0.1 (0.1, 0.4)	3.6
Garden	0.1 (0.1, 0.4)	7.7	0.1 (0.0, 3.4)	2.0
Fast food restaurant	0.0 (0.0, 0.0)	0.0	0.0 (0.0, 0.0)	0.0
TOTAL	1.83 (1.8, 2.9)	100.0	4.0 (3.3, 6.6)	100.0
**Purchaser**				
Own purchase	0.2 (0.0, 0.7)	100.0	0.7 (0.3, 1.8)	73.0
Other child	0.0 (0.0, 0.0)	0.0	0.2 (0.1, 0.6)	24.7
Other adult	0.0 (0.0, 0.0)	0.0	0.0 (0.0, 0.1)	2.3
TOTAL	0.2 (0.0, 0.7)	100.0	0.9 (0.4, 2.2)	100.0

* Cookies, cakes, desserts/puddings and pastries (baked and deep fried); ** Food retail includes supermarket, market, *fale koloa*, and other retail outlets; *** 0 (0, 0) means no food consumption or too little data to calculate the daily rate. CI: confidence interval.
